# Chitin Cryogels Prepared by Regeneration from Phosphoric Acid Solutions

**DOI:** 10.3390/ma14185191

**Published:** 2021-09-09

**Authors:** Irina V. Tyshkunova, Dmitry G. Chukhchin, Iosif V. Gofman, Ekaterina N. Pavlova, Vadim A. Ushakov, Elena N. Vlasova, Daria N. Poshina, Yury A. Skorik

**Affiliations:** 1 Institute of Macromolecular Compounds of the Russian Academy of Sciences, Bolshoi VO 31, St. Petersburg 199004, Russia; tisha19901991@yandex.ru (I.V.T.); gofman@imc.macro.ru (I.V.G.); rn-ka@mail.ru (E.N.P.); ushakovvadim@list.ru (V.A.U.); spectra@imc.macro.ru (E.N.V.); poschin@yandex.ru (D.N.P.); 2 Department of Biology, Ecology and Biotechnology, Northern (Arctic) Federal University Named after M.V. Lomonosov, Severnaya Dvina Emb. 17, Arkhangelsk 163002, Russia; dimatsch@mail.ru

**Keywords:** chitin cryogels, phosphoric acid, macroporosity, tissue engineering

## Abstract

Cryogelation is a developing technique for the production of polysaccharide materials for biomedical applications. The formation of a macroporous structure during the freeze-drying of polysaccharide solutions creates biomaterials suitable for tissue engineering. Due to its availability, biocompatibility, biodegradability, and non-toxicity, chitin is a promising natural polysaccharide for the production of porous materials for tissue engineering; however, its use is limited due to the difficulty of dissolving it. This work describes the preparation of cryogels using phosphoric acid as the solvent. Compared to typical chitin solvents phosphoric acid can be easily removed from the product and recovered. The effects of chitin dissolution conditions on the structure and properties of cryogels were studied. Lightweight (ρ 0.025–0.059 g/cm^3^), highly porous (96–98%) chitin cryogels with various heterogeneous morphology were produced at a dissolution temperature of 20 ± 3 °C, a chitin concentration of 3–15%, and a dissolution time of 6–25 h. The crystallinity of the chitin and chitin cryogels was evaluated by ^13^C CP-MAS NMR spectroscopy and X-ray diffractometry. Using FTIR spectroscopy, no phosphoric acid esters were found in the chitin cryogels. The cryogels had compressive modulus E values from 118–345 kPa and specific surface areas of 0.3–0.7 m^2^/g. The results indicate that chitin cryogels can be promising biomaterials for tissue engineering.

## 1. Introduction

Chitin is a widespread polysaccharide in Nature, the second most abundant after cellulose [1,2]. Its biocompatibility, biodegradability, renewability and antibacterial properties make it attractive for use in various fields, including the production of materials for biomedical applications [3,4,5]. One of the new directions for producing these materials is cryogelation, and freeze-dried chitin hydrogels can produce materials with heterogeneous porous structures [6]. However, the presence of strong inter- and intramolecular hydrogen bonds between the chitin polymer chains decreases the solubility of chitin in many organic solvents, thereby limiting its wide application [7].

Previously [8,9,10], ionic liquids were used as chitin solvents, and several ionic liquids were used to produce chitin/cellulose composite gels. Heating up to 100 °C also produced a composite solution with a chitin concentration of 5% and cellulose concentration of 10%. In this case, the time required for the formation of gels was 4 days [10]. Chitin can also be dissolved using LiCl/dimethylacetamide (DMAc) [11,12], alkali solutions, a mixture of NaOH and urea, thiourea [13,14,15], and methanol saturated with calcium chloride dehydrate [16,17].

The type of solvent and the conditions of dissolution affect the ultimate structure and properties of porous chitin-based materials. Materials with a density of 0.125 g/cm^3^ and a porosity of 92% were obtained by dissolving chitin in DMAc/LiCl, regenerating, and drying in supercritical CO_2_ [12]. Ultra-light porous chitin aerogels with a density of 0.039–0.063 g/cm^3^, a porosity of 84–90%, and low crystallinity as a result of the redistribution of hydrogen bonds in the chitin structure were obtained by dissolving chitin in ionic liquids, regenerating in ethanol, and then drying in supercritical CO_2_. These aerogels showed extremely low cytotoxicity with L929 fibroblasts [18].

Aerogels obtained by dissolving chitin in an aqueous KOH/urea solution and electrophoretic deposition showed a large surface area (289 m^2^/g), substantial nanoporosity (100 nm), good biocompatibility (cell viability > 90%), and high water adsorption capacity (397%) [19]. Compared to gauze and commercial wound dressings, the chitin-based aerogels showed a higher stimulatory effect on wound healing and contributed to better cytocompatibility, hydrophilicity, and air permeability. Aerogels with a density of 0.043–0.113 g/cm^3^, porosity up to 97%, surface area of 261 m^2^/g, and good mechanical properties (compressive modulus from 7 to 9.3 MPa) were produced from chitin nanoparticles obtained by hydrolysis of chitin in 3N HCl for 90 min under stirring [20]. Aerogels produced by ultrasonic treatment retained the thermal stability of the chitin nanoparticles, a feature that is important in tissue engineering. Cryogels with good mechanical strength, a density of 0.89–10.83 mg/cm^3^, and a porosity of 99.24–99.94% were also obtained using chitin nanoparticles obtained by treating chitin with 33% NaOH at 90 °C for 4 h, dispersing with a microfluidizer, crosslinking with glutaraldehyde, and subjecting to a freeze-thaw step [21].

Scaffolds based on chitin have been obtained using a solvent system of calcium chloride and methanol, followed by freeze-drying the regenerated aqueous chitin [17]. Composite scaffolds were obtained by freeze-drying an α-chitin hydrogel with bioactive glass-ceramic nanoparticles [22]. The produced materials had good swelling, mechanical strength, and high porosity and showed high biological activity.

Taken together, the available evidence indicates possibilities for the production and application of chitin-based cryogels in tissue engineering. However, the methods used previously for dissolving chitin to produce porous materials are lengthy and require special conditions. The chitin solvents used also have significant disadvantages, including the low volatility of ionic liquids, which complicates their regeneration. The significant disadvantage of using DMAc/LiCl is the difficulty of removing LiCl from the final products. When using solvents containing NaOH, Na^+^ ions penetrate deeply into the structure of the polysaccharide, so alkalis are difficult to remove. All these impurities are unfavorable for biomedical applications.

A less common solvent for chitin dissolution is concentrated phosphoric acid [23,24]. At a minimum concentration of 75%, phosphoric acid easily dissolves α-chitin at room temperature [25]. In this case, the maximum concentration of chitin in the solution was 3%. During the first hours in a phosphoric acid solution, the regenerated chitin is not chemically modified, but its molecular weight decreases. The high viscosity of the solution rapidly decreases within 12 h, and the prolonged dissolution of chitin (one week) in phosphoric acid results in the formation primarily of the monophosphate. Previous studies [25] have identified concentrated phosphoric acid as a good solvent for chitins with an average degree of polymerization from 100 to 1000, and up to 4% chitin was easily dissolved in 85% phosphoric acid. Subsequent regeneration in water produced chitin nanofiber and nanosphere structures [24]. Changing the temperature and dissolution time changed the yields and sizes of the chitin nanofibers. In the process of dissolution, the degree of phosphorylation of chitin was less than 1%, whereas the crystallinity decreased after regeneration [24].

**Scheme 1:**
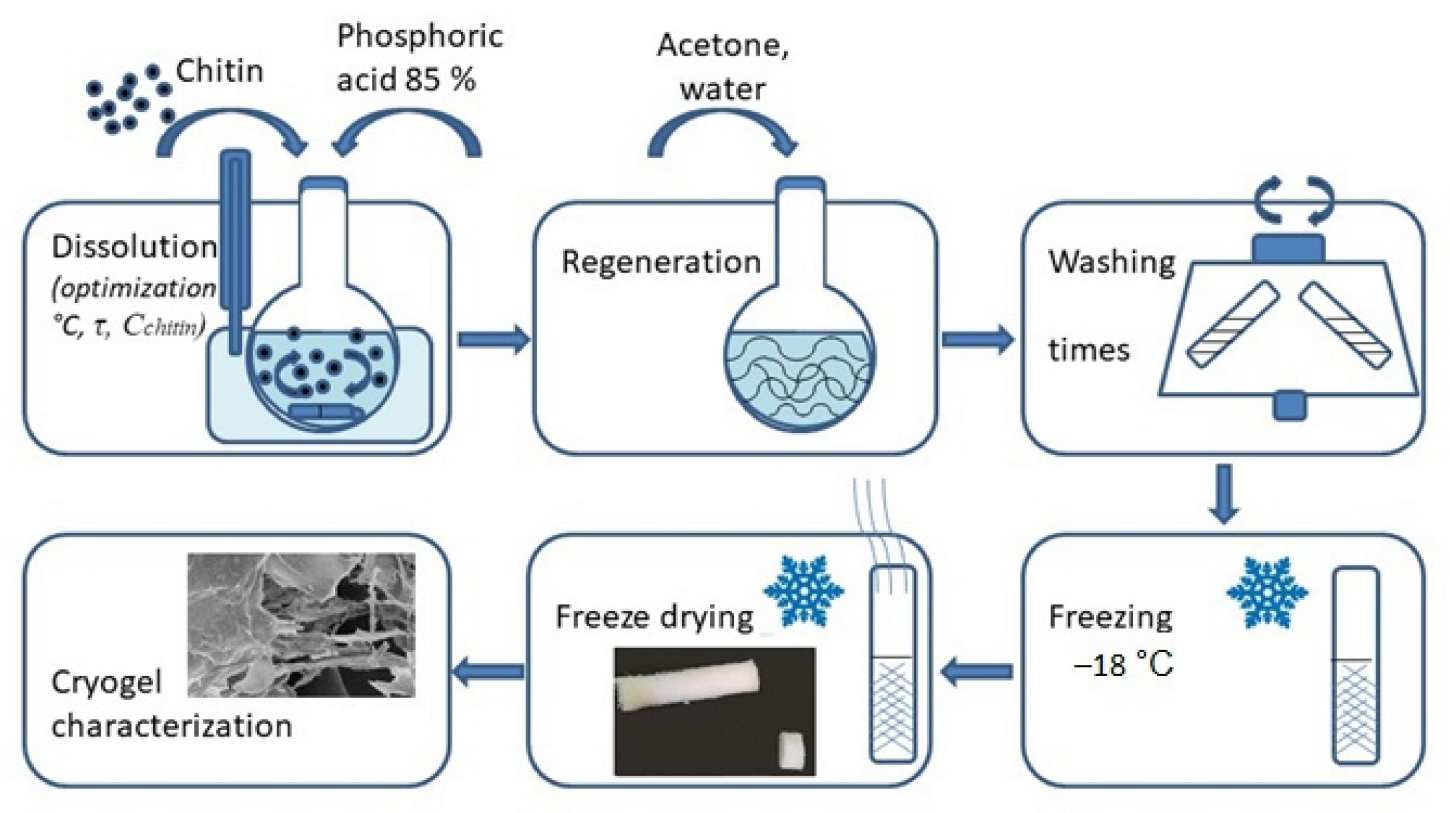
The overall structure of the work.

In the present study, the disadvantages of typical chitin solvents (ionic liquids, LiCl/DMAc, alkaline solutions, methanol saturated with calcium chloride dehydrate) were taken into account, and a method was proposed to produce cryogels after dissolving chitin in phosphoric acid. Phosphoric acid is used in the food industry (production of salts for animal feed, acidification of food and drink), in the pharmaceutical industry, for surface treatment and the production of fertilizers [26]. Phosphoric acid can be considered a “green” solvent, as it gently dissolves chitin [23] and can be easily removed, regenerated, and reused. This makes phosphoric acid a technologically superior and easy to use solvent. The dissolution process of chitin in phosphoric acid was previously studied [23,24,25]; however, no attempt was made to produce chitin cryogels using phosphoric acid as a solvent. Here, the effects of the production process parameters on the structure and properties of the resulting chitin cryogels were investigated. Scheme 1 illustrates the overall structure of the work.

## 2. Materials and Methods

### 2.1. Materials

The crab α-chitin (Biolog Heppe GmbH, Landsberg, Germany) used had a weight average molecular weight (M_w_) of 5.86 × 10^5^ and dispersity (Ð) of 4.3. A degree of acetylation of 0.98 was calculated using solid-state cross-polarization magic angle spinning carbon-13 nuclear magnetic resonance (^13^C CP-MAS NMR) spectroscopy. Phosphoric acid 85 wt.% in H_2_O (99.99% trace metals basis), acetone (purity of 99.7%) and DMAc (purity of 99.95%) were supplied by Vekton (St. Petersburg, Russia). Lithium chloride (purity of 99%) was supplied by Sigma-Aldrich (St. Louis, MO, USA).

### 2.2. Preparation of Chitin Cryogels

Chitin solutions (3.0–15.0 wt.%) were prepared using 85% H_3_PO_4_. Chitin powder was mixed with H_3_PO_4_ under various conditions (different chitin concentrations and dissolution times, Table 1). The solutions (5 mL) were periodically stirred until the complete dissolution of chitin (transparent solutions). Acetone was used as a precipitant for chitin from phosphoric acid solutions. The regenerated chitin was washed with distilled water via several cycles of centrifugation (4500 rpm for 5 min) until neutral pH, frozen at −18 °C, and freeze-dried in a 10 N freeze-dryer (Shanghai Drawell Scientific Instrument Co., Shanghai, China).

### 2.3. General Methods

High performance size-exclusion chromatography was performed on a Prominence LC-20 system (Shimadzu, Kyoto, Japan) consisting of a parallel double micro plunger pump (LC-20AD), a degassing unit (DGU-20 A3R), a 20 μL manual injector (Rheodyne 7725i), a column oven (CTO-20A), and a refractive index (RID-20A) detector. Chromatographic separation was carried out using two GRAM columns (PSS, Mainz, Germany) (1000 Å, 300 × 8 mm) and a GRAM precolumn (50 × 8 mm). The column oven and refractive index detector were maintained at 70 °C and 55 °C, respectively. Chitin samples were prepared according to the method previously described for cellulose [27] and eluted at a flow rate of 1.0 mL/min with a mobile phase of 0.5% LiCl in DMAc. The detector was calibrated with a pullulan standards kit with M_w_ ranging from 345 to 805,000 (PSS, Mainz, Germany). Data were acquired using LC-Solution software (version 5.71 SP1), while LabSolutions GPC software (version 5,93, 2018, Shimadzu, Kyoto, Japan) was used for molecular weight calculations. Note that the molecular weights determined from size-exclusion chromatography are the apparent molecular weights relative to pullulan standards.

The cryogel yield, geometrical dimensions, volume shrinkage, volume variation (∆V), density (ρ), specific mass, porosity (the density of bulk chitin is taken as 1.425 g/cm^3^ [28], and swelling were measured and calculated as described previously [29].

The ^13^C CP-MAS NMR spectra were recorded on a Bruker AVANCE II-500 WB instrument (Bruker, Billerica, MA, USA). The degree of acetylation of chitin was determined from the ratio of the integral intensities of the C1 signal and the CH_3_ group signal [30].

X-ray diffraction was performed using a DRON-3M instrument (Burevestnik, St. Petersburg, Russia) at a radiation wavelength of Cu Kα λ = 1.54 Å. The crystallinity of the chitin and chitin cryogels was calculated as the ratio of the intensity of the (110) reflex to the amorphous halo at 2θ = 16° [31].

FTIR spectra were obtained using a Vertex-70 FTIR spectrometer (Bruker) equipped with a ZnSe attenuated total reflectance accessory (PIKE Technologies, Fitchburg, WI, USA). When analyzing the spectra, a correction was introduced for the depth of penetration of the beam into the sample depending on the wavelength. FTIR and NMR spectra were processed using MestReNova software (version 6.0.2-5475, 2009, Mestrelab Research S.L., Santiago de Compostela, Spain).

The morphology of the chitin cryogels was studied using a SIGMA VP field emission scanning electron microscope (ZEISS Research Microscopy Solutions, Jena, Germany) at 10 kV accelerated voltage. The samples were coated with 5-nm Pt/Pd metal layer using a Q150TES sputtering system (Quorum, Laughton, East Sussex, UK).

The specific surface area was determined using a QuadraSorb instrument (Quantachrome Instruments, Boynton Beach, FL, USA) and the Brunauer, Emmett, and Teller approach. The Kr adsorption isotherm were measured at 77 K after degassing of the samples at 70 °C.

The mechanical properties of the chitin cryogels were studied on an AG-100kN X Plus setup (Shimadzu, Kyoto, Japan). The test samples were prepared in tablet form with a height and diameter of about 1 and 2 cm, respectively. The compression was performed at room temperature (20 ± 3 °C) with a compression rate of 5 mm/min. All experiments were performed in triplicate, and the data are displayed as mean ± standard deviation.

## 3. Results and Discussion

### 3.1. Chitin Dissolution in 85% Phosphoric Acid and Preparation of Chitin Cryogels

Previous work confirmed that chitin at concentrations up to 4% was easily dissolved in concentrated phosphoric acid [24]. Initially, we tested the production of cryogels from a solution with a chitin concentration of 1%. In this case, a powder was obtained, and some fragments of cryogel were visible. Therefore, we decided to use chitin concentrations from 3 to 15% (Table 1), similarly to the previously obtained cellulose cryogels [29,32].

The production of transparent solutions was assumed to correspond to the complete dissolution of chitin in phosphoric acid. The use of water as a precipitant led to the complete dissolution of chitin at the phosphoric acid washing stage. Therefore, we used acetone to precipitate the chitin from the phosphoric acid solutions. The regenerated chitin was washed free of residual phosphoric acid using water, and then frozen and freeze-dried. Cryogels that retained the shape of the test tubes in which the freezing and freeze-drying took place were obtained from all solutions except Sample 7.

We obtained 5% solutions of chitin in 16 h at room temperature (20 ± 3 °C). Small fragments of chitin were still visible in the solution after 6 h of dissolution (Sample 5). We obtained 10 and 15% solutions of chitin in 24–25 h at 20 ± 3 °C; this was a shorter time than the 48 h required for 8.4% cellulose solution at 20 ± 2 °C [29]. The solution with a 15% chitin concentration of was difficult to stir at the initial stages of dissolution. After 6 h of dissolution, the stirring improved.

The lowest cryogel yield was obtained for Sample 1 (Table 1). This is explained by the easier access of the solvent to the chitin polymer chains at low concentrations. A lower yield (45.4%) was also obtained for Sample 7. Increasing the chitin dissolution time to 48 h led to the formation of a powder that did not form a cryogel. Samples 2–4 gave cryogel yields of about 77%. Sample 5 gave a smaller yield (66.8%) due to losses at the washing stage during regeneration of the chitin from phosphoric acid and due to incomplete dissolution of chitin in acid after 6 h. The highest yield (81.2%) was obtained for Sample 6 (the dissolution time for chitin was 16 h).

The cryogels with the lowest density (Samples 1 and 5) had the highest porosity, at 98%. Increasing the chitin concentration resulted in a lower porosity (96–97%) and a higher density (ρ 0.04–0.06 g/cm^3^) (Samples 2–4). Chitin cryogels were less dense than the previously obtained cellulose cryogels (ρ 0.091–0.161 g/cm^3^) using phosphoric acid as a solvent [29]. Only cryogels based on nanochitin had a lower density (ρ 0.89 ± 0.22 and 1.28 ± 0.24 mg/cm^3^) [21]. Notably, the density of the obtained cryogels was similar to the density for previously obtained chitin aerogels (ρ from 0.0048 to 0.100 g/cm^3^) [12,20,28,33].

The M_w_ of chitin decreased after dissolution in phosphoric acid and regeneration (Table 1). The smallest value was obtained for Sample 7 (powder). The M_w_ of Sample 1 was 41,000, which is sufficient to obtain a cryogel. The highest M_w_ was obtained for Sample 5 (Table 1). After 6 h of dissolution in phosphoric acid, chitin showed less destruction. The M_w_/M_n_ ratio (or the dispersity, Ð) is a measure of the nonuniformity of the polymer sample. A Ð value close to 1 indicates a narrow molar mass distribution, while higher values reflect increasing non-uniformity (i.e., broad or stretched molar mass distributions). The dispersity increased with an increase in the chitin concentration or a decrease in the dissolution time of chitin when producing cryogels (Table 1).

The regeneration of chitin from solutions of phosphoric acid and the subsequent freezing and freeze-drying caused a volume shrinkage of the cryogel samples when compared with the volume of the initial solution. The greatest shrinkage was noted for Sample 1 (33.5%) and was associated with the lowest concentration of polysaccharide in solution and the lowest cryogel volume (3.3 cm^3^). Increasing the chitin concentration in solution up to 5% reduced this volume shrinkage to 26% for Sample 2 (cryogel volume 3.7 cm^3^) and to 3.9% for Sample 6 (cryogel volume 4.8 cm^3^). Samples 3, 4, and 5 did not show shrinkage, and their cryogels had a larger volume (7.8, 9.7, and 6.8 cm^3^, respectively) compared to the initial volume of the solutions (5 cm^3^).

The chitin hydrogels (washed, regenerated chitin) were characterized by determining the volume variation (the ratio of the hydrogel volume to the volume of the initial solution). In this case, a negative value corresponds to shrinkage, while a positive value corresponds to chitin swelling after regeneration. Positive results were obtained for all samples (Table 1). An increase in the chitin concentration (Samples 1–4) also increased the volume of the swollen hydrogel. Samples 3 and 4 had the largest values for volume variation.

Thus, to obtain chitin cryogels, a dissolution time of 16–25 h is sufficient. During this time, solutions with a chitin concentration from 5 to 15% in phosphoric acid can be obtained. This confirms the better solubility of chitin in comparison with the previously studied cellulose [29].

### 3.2. Swelling in Water of Chitin Cryogels

The degree of swelling of the hydrogels (the ratio of the mass of the original chitin and the mass of the obtained hydrogel) was also evaluated (Table 1). A higher chitin concentration resulted in a lower swelling, in agreement with previously obtained results for cellulose cryogels [29].

The stability of chitin cryogels in water was determined at room temperature (20 ± 3 °C). After storage in water for 24 h, the samples were weighed. The swelling ratios (SR) are shown in Table 1. Sample 1 fell apart during weighing. Samples 2–6 were stable in water. A lower sample density gave a higher SR value. Sample 5 had the lowest density and the highest SR value.

### 3.3. Specific Surface Area of Chitin Cryogels

The specific surface area of chitin cryogels ranged from 0.3 to 0.7 m^2^/g (Table 1). A previous study [21] on cryogels based on nanochitin showed a decrease in the specific surface area from 25.3 to 12.2 m^2^/g with an increase in the nanochitin content from 0.2 to 0.6%. In the present case, samples with chitin concentrations of 3 and 5% gave approximately the same results for the specific surface area (0.3 m^2^/g). An increase in the chitin concentration to 10% led to an increase in the specific surface area to 0.66 m^2^/g (Sample 3). A further increase in the chitin concentration (15%) resulted in a twofold decrease in the specific surface area (Sample 4). Notably, the specific surface area was lower for the chitin cryogels than for cellulose cryogels obtained using the same methodology (from 1.03 to 1.77 m^2^/g) [29].

### 3.4. Crystallinity of Chitin and Chitin Cryogels

**Figure 1:**
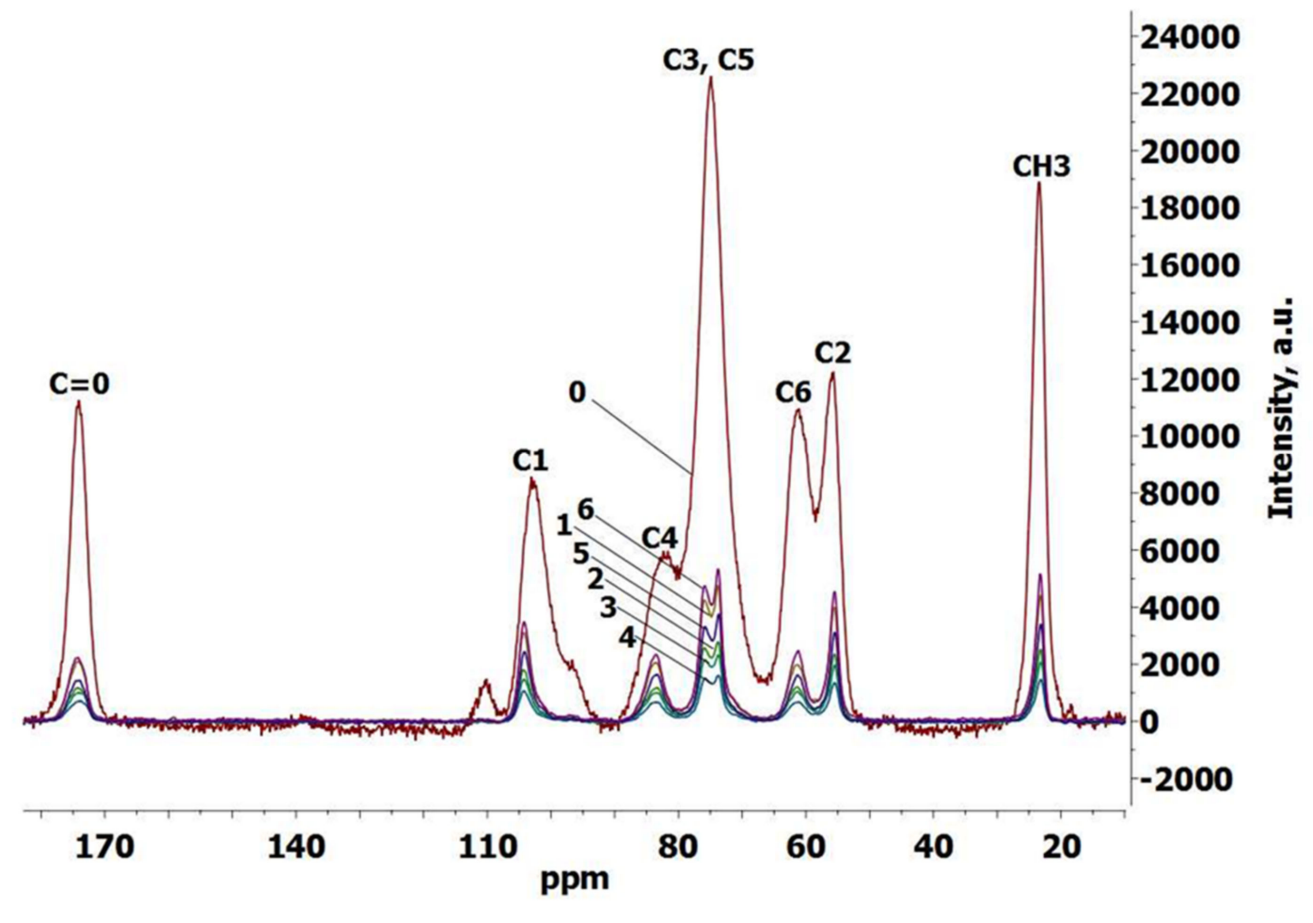
^13^C CP-MAS NMR spectra of chitin and chitin cryogels: 0 = chitin, 1–6 = chitin cryogel samples according to Table 1.

The method for determining the crystallinity of chitin by ^13^C CP-MAS NMR spectroscopy is not described. In contrast to cellulose [29], the spectra of chitin showed no separation of signals from C4 (82.4 ppm), C6 (61.1 ppm), or C3 and C5 (74.9 ppm). Changes in the spectrum of chitin after dissolution in phosphoric acid (chitin cryogel spectra) are shown in Figure 1. Chitin cryogels show a separation of signals from the C3 and C5 atoms, whereas the original chitin showed no separation. Separation of the C3 and C5 signals has been described in the literature as a characteristic of α-chitin [34], whereas these signals are not separated in the β-chitin spectrum. These two allomorphic forms of chitin differ in the configuration of their space group and crystal lattice, which is determined by the orientation of the polysaccharide chains and the H-bonding pattern. α-Chitin is more widespread in nature; it is characterized by antiparallel chains and a strong network of intra- and inter-molecular H-bonds, including bonding between sheetlike molecular structures [35]. Due to its supramolecular structure, α-chitin has high stability; it is found mostly in the rigid tissues of crabs and insects. By contrast, β-chitin is derived from the flexible structures of squid pen and pogonophora tubes and has a parallel chain orientation but weak intersheet bonding. Therefore, it shows greater swelling ability and reactivity compared to α-chitin [36].

Tanner et al. [37] demonstrated that the highly crystalline β-chitin from diatoms also showed separation of the C3 and C5 signals in ^13^C CP-MAS spectra. The crystalline samples gave narrow signals that were used for accurate determination of β-chitin chemical shifts and for signal assignment in spectra of more amorphous β-chitin samples [38], where signal broadening and merging occurred. Thus, ^13^C CP-MAS spectra are not sufficient for the correct determination of chitin allomorph modifications [36,37].

Another difference between the spectra of α- and β-chitin is in the acetyl carbon signal at approximately 174 ppm. In the case of α-chitin, the deconvolution of carbonyl signal allowed determination of the carbonyl groups involved in double and single H-bonding [30]. In the case of β-chitin, carbonyl peak asymmetry is believed to arise from coupling with the ^14^N nucleus [37]. In our case, the peaks in the original chitin spectrum were rather broad, which precluded a clear distinguishing of the crystalline modification. Since only the α-chitin structure has been described for crab chitin, we assume that our original chitin is a highly amorphous sample with a destroyed H-bond network. The cryogels did show a separation of signals from the C3 and C5 atoms, becoming more similar to the crab α-chitin spectra described in the literature [34] and indicating crystallinity changes after regeneration from phosphoric acid. Notably, the degree of chitin acetylation did not change after dissolution in phosphoric acid and regeneration with acetone.

**Figure 2:**
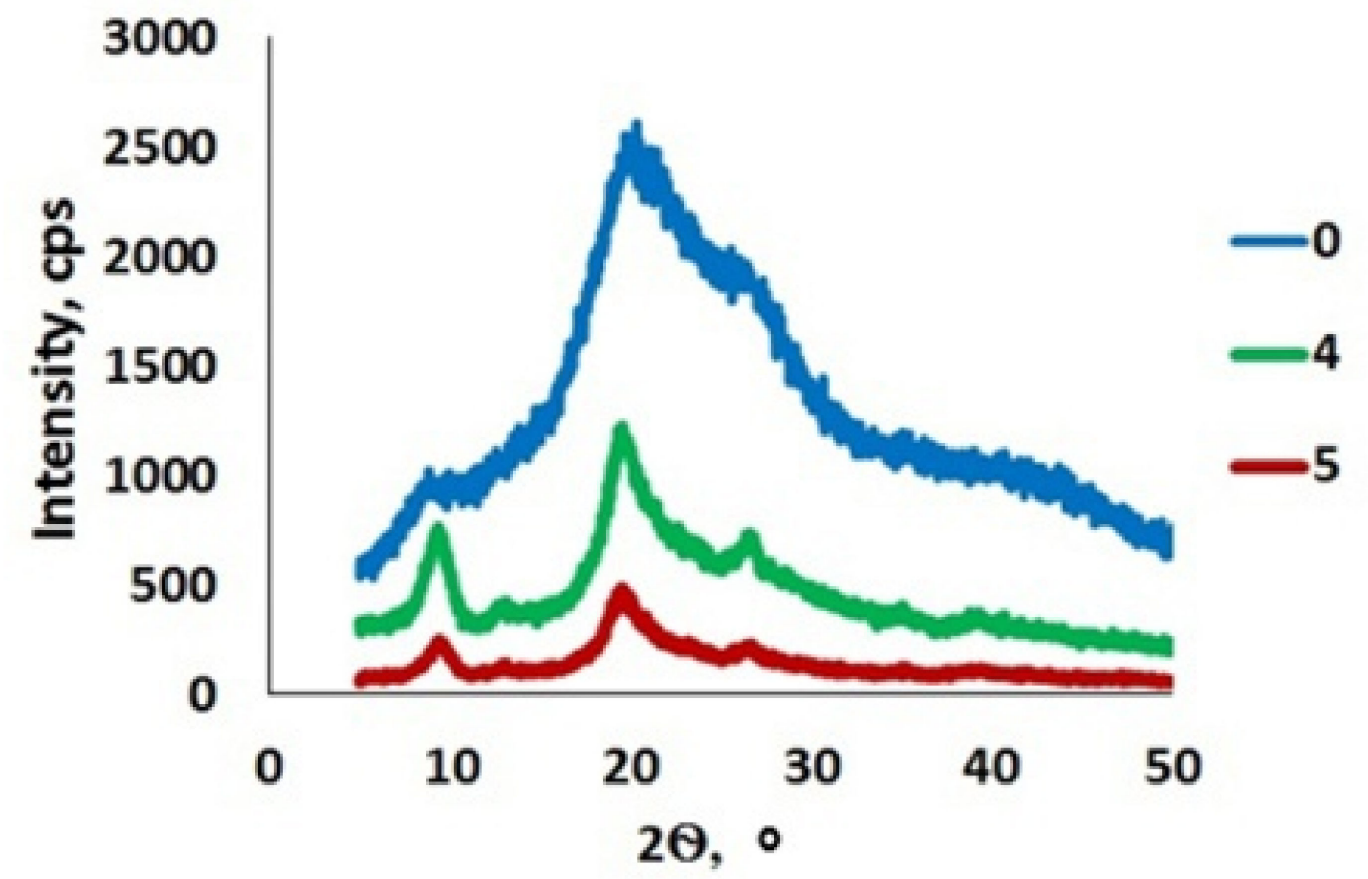
X-ray diffraction patterns of the original chitin (0) and chitin cryogel samples 4 and 5.

We determined the crystallinity of the original chitin sample and chitin cryogels by X-ray diffraction; the X-ray diffraction diagrams for chitin and several chitin cryogel samples are presented in Figure 2. We chose a cryogel obtained with the shortest chitin dissolution time (Sample 5) and a cryogel obtained from a solution with the maximum chitin concentration (Sample 4). According to the literature, both α- and β-chitin show two sharp diffraction peaks at 2θ of approximately 8.5–9.5° from the (020) planes and 18.5–19.5° from the (110) planes of crystalline unit cells, as well as some weak peaks [31]. Kaya et al. [34] observed (020) reflection for α- and β-chitin at 9.46° and 8.59°, respectively. For α-chitin, a sharper second reflection peak was observed at 12.74°, while the same peak for β-chitin was slightly weaker and appeared at 12.29° [34]. In our case, the (020) crystalline reflection was observed at 8.84° for chitin and at 9.14° (Sample 4) and 9.28° (Sample 5) for cryogels. For chitin cryogels, the second reflection peak was observed at 12.48° (Sample 4) and at 12.8° (Sample 5). For the original chitin, determining a clear peak was difficult.

Therefore, the main difference in the XRD diagrams of the original chitin and the obtained cryogels was related to a narrowing of the crystalline reflections due to an increase in the crystallinity of chitin after treatment with phosphoric acid and the subsequent stage of regeneration. The crystallinity of chitin was estimated as 58%, while for cryogels the crystallinity increased to 85%. This difference was rather large, indicating significant crystallinity changes during regeneration. For crystallinity assessment, we used the approximate evaluation involving a total intensity at 2θ of 20° and an intensity of amorphous scattering at 16°. This crude estimation has recently been shown to be inaccurate [39]; however, it is easy and does not require any crystalline and amorphous reference samples.

### 3.5. FTIR Spectroscopy

**Figure 3:**
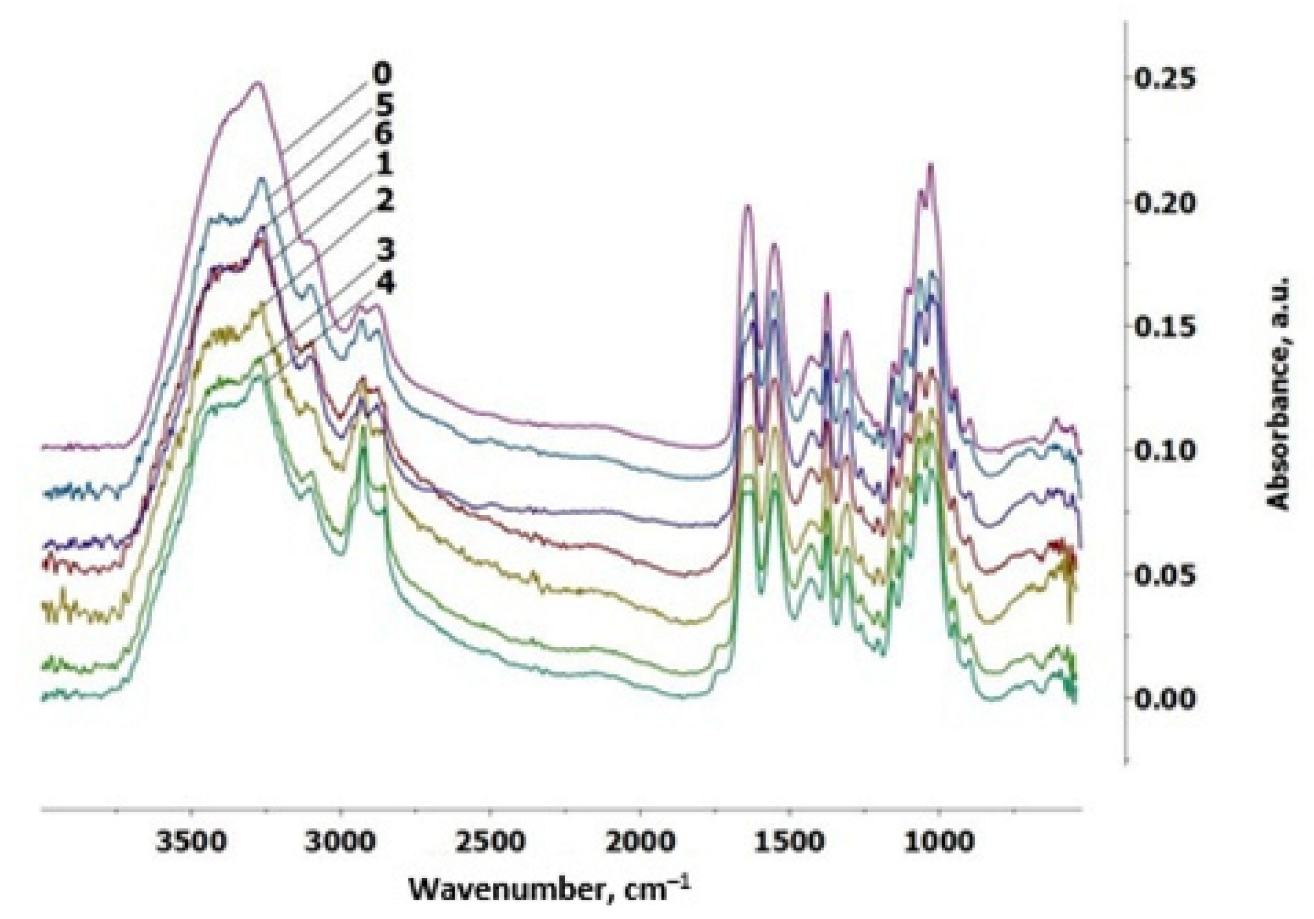
FTIR spectra of chitin and chitin cryogels; 0 = original chitin, 1–6 = samples of chitin cryogels according to Table 1.

The FTIR spectra of the original chitin and chitin cryogels were similar (Figure 3). All spectra had characteristic chitin functional groups, including an NH stretching vibration band at 3269 cm^−1^, an amide I band at 1640 cm^−1^, and an amide II band at 1552 cm^−1^. This indicated that the chemical structure of chitin did not change after dissolution in phosphoric acid and regeneration with acetone.

The most obvious spectral difference between α- and β-chitin described in the literature is the frequency of the vibration modes of amide I in the region 1660–1620 cm^−1^ (two absorption bands appear with α-chitin, but only one band with β-chitin) [40]. However, for highly crystalline β-chitin, a two-band pattern in the region of 1660–1620 cm^−1^ has also been reported [41]. Chitin cryogels were characterized by two absorption bands at 1646–1650 cm^−1^ and at 1625–1629 cm^−1^. The original chitin had two absorption bands at 1659 cm^−1^ and 1625 cm^−1^. Poor band separation compared to cryogels may be due to the higher water content.

Two bands in α-chitin characterize two types of amides: half of the carbonyl groups are hydrogen bonded to the amino group within the same chain (C = O…H–N), and this is responsible for the vibration mode at 1650 cm^−1^. The remaining carbonyl groups give the same bond, plus one more with the –CH_2_OH group from the side chain, which appears as a band at 1629 cm^−1^. The existence of these interchain bonds is responsible for the high chemical stability of the α-chitin structure.

In the region of the OH and NH groups (3600–3000 cm^−1^), α-chitin has a more detailed structure than β-chitin due to the different H-bond pattern [38]. The bands in the region of 3340–3440 cm^−1^ reflect intramolecular hydrogen bonds with the participation of OH(6)…O=C, O(3)H…O(5) from the ring. The bands at 3269 cm^−1^ and 3097 cm^−1^ characterize NH vibrations of the amide (intermolecular hydrogen bond C=O…H–N and the NH groups intramolecularly bonded by H, respectively). In our case, the differences in the region of the OH and NH groups were not significant, supporting the conclusion that the original chitin was in a highly amorphous α-chitin state and that the crystallinity of the samples should be considered when interpreting the ^13^C CP-MAS and IR spectra.

Previously, the phosphorylation of chitin was confirmed by the appearance of a broad peak at 3428 cm^−1^ (P–OH group), a peak at about 1221 cm^−1^ (asymmetric stretching of the phosphate group (P=O), a small peak at 975 cm^−1^ (P-OH), a small peak at 800 cm^−1^, and a peak at 1050 cm^−1^ (stretching C–O–P) [42]. However, these bands were absent from the FTIR spectra of the cryogels (Figure 3), indicating that, during the regeneration with acetone and washing with water, the phosphate groups were completely hydrolyzed, in agreement with what we have observed for cellulose [29].

### 3.6. Mechanical Properties

**Figure 4:**
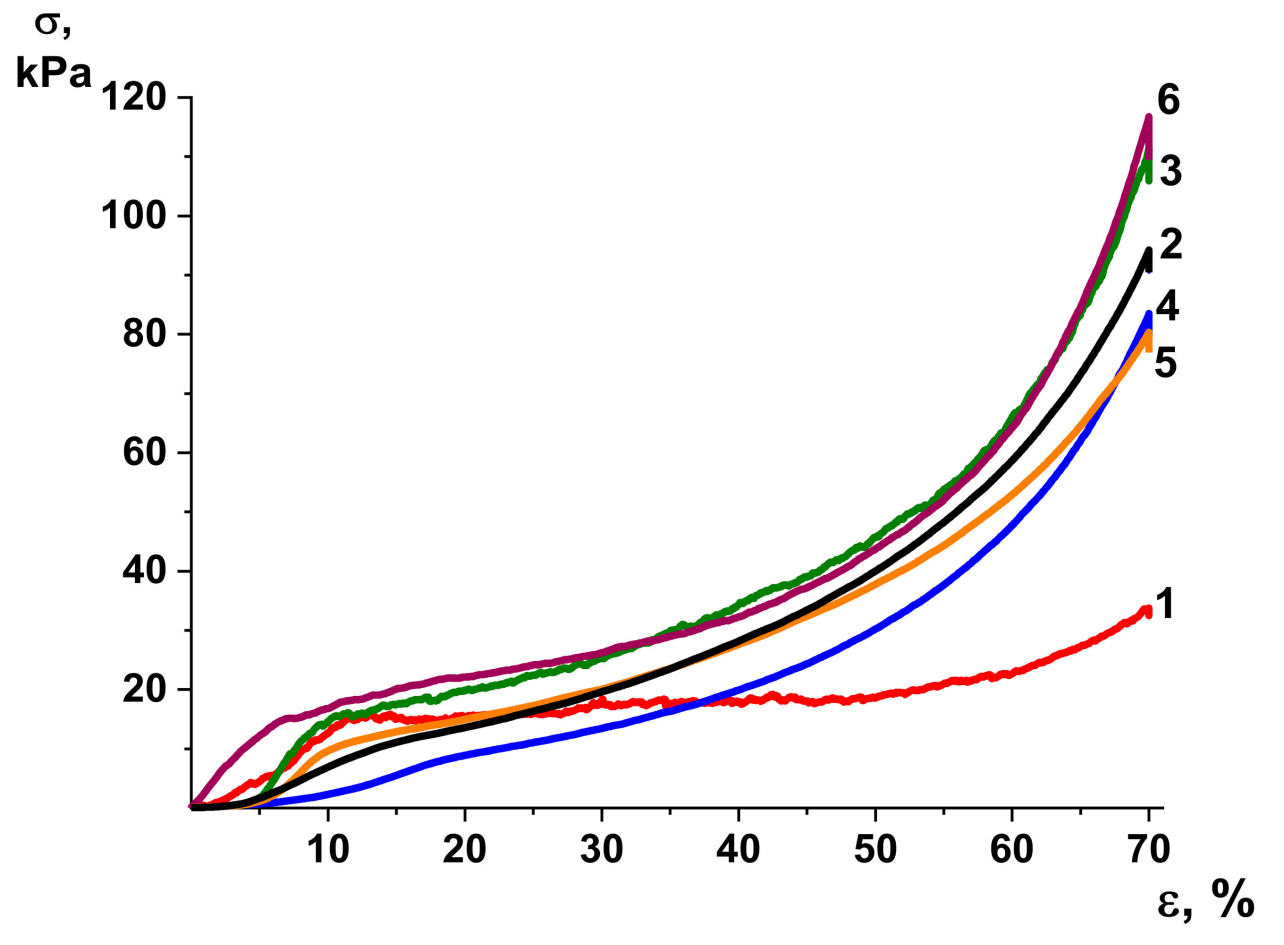
Stress–strain curves for chitin cryogels (Samples 1–6).

Strength is an important quality of cryogels for tissue engineering applications [43,44]. Compressive modulus (E), yield stress (σ_y_), and stress corresponding to the critical deformation of 70% (σ_max_) were determined for chitin cryogels. Note that recording the moment of destruction of the samples was not possible during the compression testing. The stress-strain curves did not show a sharp decrease in stress, which could indicate destruction of the material. Therefore, the compression strain limit was set at 70%. The mechanical parameters are presented in Table 2, and the types of deformation curves are shown in Figure 4.

The shape of the stress–strain curves was similar for all the cryogel samples. The lowest compressive modulus E was obtained for the cryogel sample with the lowest M_w_ (Sample 1). Samples 2, 5, and 6 (chitin concentration 5%) were characterized by a decrease in the M_w_ of chitin with an increase in dissolution time, and, accordingly, a decrease in the modulus E (Table 2). Sample 3 had the highest modulus E.

Thus, an increase in the chitin M_w_ increased the strength of the cryogel, in agreement with previous findings for cellulose cryogels [29]. However, the chitin cryogels are not as strong as cellulose cryogels obtained with the same methodology (for cellulose, the compressive modulus E is from 330 to 3675 kPa) [29].

### 3.7. SEM Morphology of Chitin Cryogels

**Figure 5:**
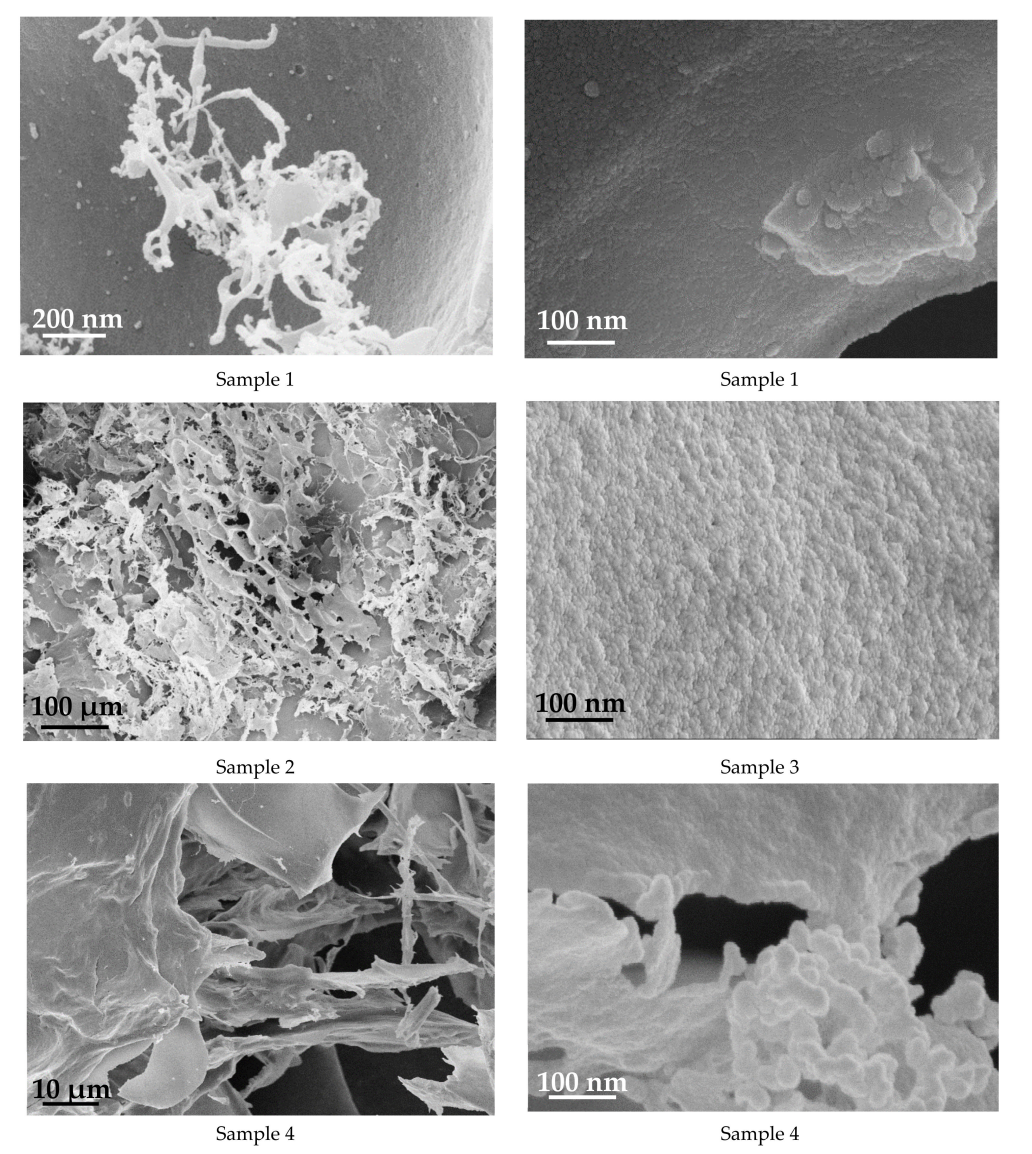
SEM images of chitin cryogels (Samples 1–4).

All cryogels had a similar morphology. Both film and network structures were found, consisting of cauliflower-like structures formed by clusters of small spheres (examples are shown in Figure 5). The chitin cryogels had a similar morphology to that of previously obtained nanochitin cryogels [21] and chitin aerogels [19,28,45]. The chitin aerogels with complex hierarchical morphology accelerated the healing of wounds [19]. Cell studies have shown that fibroblast cells (L929 cell line) are well attached onto chitin aerogels and maintain their normal morphologies compared with controls in normal culture plates, indicating a certain biocompatibility of the chitin aerogels [28]. 

The physical properties and morphology of nanochitin cryogels were deemed suitable for advanced biobased materials [21]. Therefore, chitin cryogels are good candidate scaffolds for tissue engineering because of their complex hierarchical morphology.

## 4. Conclusions

Ultralight (0.025–0.059 g/cm^3^), highly porous (96–98%) chitin cryogels were obtained using phosphoric acid as a solvent. The influence of the dissolution time and the polysaccharide concentration on the properties of cryogels was estimated. Cryogels could not be produced from a solution with a chitin concentration of less than 3%, whereas obtaining a solution with a chitin concentration of 15% was difficult in the first stages of the dissolution process due to the complexity of mixing. Dissolving chitin in phosphoric acid is a simple process compared to dissolving cellulose, and a dissolution time of 16–25 h at 20 ± 3 °C was sufficient to obtain 5–15% solutions. The use of acetone is necessary to precipitate chitin from phosphoric acid solutions. The cryogel with the greatest durability and stability in water and the highest specific surface area was a cryogel obtained from a 10% chitin solution. The decrease in the chitin M_w_ (to 41,000) after dissolution in phosphoric acid and regeneration with acetone did not prevent the formation of cryogels.

Analysis by ^13^C CP-MAS NMR spectroscopy, FTIR spectroscopy, and X-ray diffraction showed that, after dissolution in phosphoric acid, chitin undergoes the α-modification and that crystallinity increased significantly. No phosphorylation was evident.

All chitin cryogels had a multifarious, heterogeneous morphology ranging from film and network structures to spherical structures and aggregates of spherical structures. The morphology is similar to that previously described for nanochitin cryogels and chitin aerogels prepared from various solvents. This morphology makes cryogels good candidates for tissue engineering scaffolds.

Thus, the optimal parameters for the production of light, highly porous chitin cryogels with various heterogeneous morphology were determined as a dissolution temperature of 20 ± 3 °C, concentration of chitin in solution from 5%, and a dissolution time of 16–25 h. The molecular weight of chitin decreases significantly upon dissolution in phosphoric acid; however, this does not preclude the production of shape-retained cryogels.

The dissolution of chitin in phosphoric acid and regeneration with acetone allows the production of cryogels with different mechanical properties and porosities. The properties of the produced cryogels can be changed by varying the dissolution conditions for chitin. The resulting chitin materials can find applications in tissue engineering due to their biocompatibility, biodegradability, and nontoxicity.

## Figures and Tables

**Table 1:** Dissolution conditions and characteristics of chitin cryogels prepared from 3–15% chitin solutions in 85% phosphoric acid at 20 ± 3 °C.

Sample	Chitin Concentration, %	Dissolution Time, h	Sample Shape	Yield, %	Cryogel Volume, cm^3^	Volume Shrinkage, %	∆V, %	ρ, g/cm^3^	Porosity, %	$Q_{max},$%	M_w_ (Ð) *	SR, g/g	Specific Surface Area, m^2^/g
1	3	24	Intact cylindrical	60.7	3.3	33.5	20	0.027	98.1	6388	41,000 (4.6)	Sample fall apart	0.334
2	5	24		77.6	3.7	26.0	74	0.050	96.3	4416	73,000 (7.3)	7.2	0.292
3	10	24		77.4	7.8	−56.0	148	0.049	96.5	3091	80,450 (7.8)	7.8	0.660
4	15	25		76.4	9.7	−93.3	170	0.059	95.8	2282	63,830 (7.4)	7.2	0.330
5	5	6		66.8	6.8	−35.6	90	0.025	98.3	5737	274,600 (15.3)	16.4	-
6	5	16		81.2	4.8	3.9	90	0.042	97.0	4610	78,980 (8.2)	10.0	-
7	5	48	Powder	45.4	-	-	-	-	-	-	10,140 (2.0)	-	-

* Ð = M_w_/M_n_ is the dispersity, where M_w_ is the weight average molecular weight and M_n_ is the number average molecular weight.

**Table 2:** Mechanical parameters of chitin cryogels, mean ± standard deviation, *n* = 3.

Sample	E, kPa	σ_y_, kPa	σ_max_, kPa	ε_max_, %
1	118 ± 6	11 ± 3	33 ± 4	70
2	170 ± 21	10 ± 1	87 ± 7	70
3	345 ± 23	15 ± 3	112 ± 22	70
4	154 ± 37	9 ± 2	83 ± 16	70
5	230 ± 7	11 ± 1	80 ± 12	70
6	214 ± 23	18 ± 7	116 ± 21	70

## Data Availability

The data are contained within the article.
